# Large Duodenum

**Published:** 1915-08

**Authors:** 


**Large Duodenum** (which the author terms megaduodenum and which ought to be termed megadodekalodactylos) R. W. Corwin, Peublo, Col. Med., May 1915. The patient was a married woman, aged 29, with no significant family or personal history, except supra-orbital neuralgia and distress in stomach. April 14, 1914, abdominal exploration showed an extensive veil over the intestines, stomach, appendix and other organs. The duodenum was at first mistaken for the stomach on account of its size which exceeded that of the stomach. The appendix was removed on account of kinks and adhesions and posterior gastro-enterostomy was performed. Contrary to precedent, the author reports no permanent improvement, exacerbations occurring mainly at times of over exertion. Various authorities consulted by letter agreed as to the rarity of the condition. Huntington considers the cause as not congenital but due to pressure by the mesenteric vessels at the termination of the duodenum. L. L. McArthur adds that dragging down of these vessels by inflammatory processes in the lower abdomen and sometimes as the result of gastro-enterostomy is a special factor. Joseph C. Bloodgood adds to this group of cases the association of acute dudoenal dilatation with that of the stomach after operations, in acute typhoid, pneumonia, sepsis, acute rheumatism, etc., and also (compensatory?) dilatation of the duodenum after gastrectomy and gastro-enterostomy. Lavage and other medical treatment failing, most authorities agree on establishing a fistula between the duodenum and jejunum. Ora S. Fowler of Denver reported a personal case very similar, except that the stomach and duodenum formed a continuous cavity, due to pelvic inflammation with a series of operations. He also mentioned one due to treitz hernia, the duodenum being less than 3 inches in diameter. (Note: We have seen only one case in personal practice, diagnosed by auscultatory percussion and fluoroscope as hour-glass contraction of the stomach and, from other signs, as due to cancerous lesion. This diagnosis was mechanically correct but the cancer was at the pylorus and what was supposed to be the distal part of an hour-glass stomach was the dilated duodenum. The cause of the dilatation of the duodenum was not obvious; although there was the temptation to imagine that we saw a tendency to pressure by the vessels as mentioned. The stomach itself, contrary to what might be expected but what is not especially liable to occur, was not markedly dilated).

